# A Case of Persistent Asthma Resistant to Available Treatment Options: Management Dilemma

**DOI:** 10.7759/cureus.4194

**Published:** 2019-03-06

**Authors:** Hasnan M Ijaz, Waliul Chowdhury, Muhammad Uzair Lodhi, Qamar Gulzar, Mustafa Rahim

**Affiliations:** 1 Internal Medicine, Raleigh General Hospital, Beckley, USA; 2 Internal Medicine, West Virginia University School of Medicine, Morgantown, USA

**Keywords:** asthma treatment, severe asthma, neutrophilic asthma

## Abstract

Asthma affects nearly 300 million people worldwide, with 250,000 associated deaths annually. An estimated 5%-10% of patients have severe asthma, while only 1%-2% presented with treatment-resistant or refractory asthma. Currently, the endotype of asthma is divided into T-helper type 2 (Th2) high and Th2-low inflammation endotypes. The Th2-high endotype is characterized by eosinophilic asthma, while the Th2-low endotype is associated with neutrophilia and a pauci-granulocytic profile. The Th2-low endotype carries a high resistance to corticosteroid and bronchodilator therapy, and these patients typically have a severe and acute-onset of symptoms. We present a 57-year-old nonsmoking female with recurrent intensive care unit (ICU) admissions for severe acute asthma exacerbations, resistant to bronchodilator and steroid treatment, requiring mechanical ventilation. Currently, the guidelines for treating neutrophil-predominant Th2-low inflammation asthma have not been established. This creates a management dilemma when encountered with such a patient in clinical practice. We aim to propose targeted treatment options for these severe and potentially fatal asthma patients, with reference to current literature.

## Introduction

Asthma is an inflammatory process which reversibly obstructs small airways, leading to bronchial hyper-responsiveness. Asthma affects 300 million people worldwide with an annual death rate of 250,000 per year. Most of the patients with asthma are symptomatically controlled with currently available treatment options. However, 1%-2% of patients are treatment-resistant with severe refractory asthma. After an acute exacerbation, an estimated 30% of patients get admitted to the intensive care unit (ICU) with a mortality rate as high as 8%. Approximately 4% of patients require mechanical ventilation due to severe asthma. Severe asthma patients who get admitted to the ICU have a higher risk of death [1-4]. We present a 57-year-old female with multiple ICU admissions due to severe treatment-resistant asthma exacerbations.

## Case presentation

A 57-year-old nonsmoking Caucasian female was brought to the hospital by her husband due to sudden shortness of breath. Upon admission, the patient received nebulizer treatment and intravenous steroids, but her condition continued to deteriorate and required an urgent transfer to the ICU, in which she was placed on mechanical ventilation. The patient presented a few times similarly in the past couple of months, requiring mechanical ventilation every visit. According to the patient's husband, the patient had no sick contact, recent travel, or a productive cough before admission. He also stated that she did not complain of chest pain or shortness of breath upon exertion.

Upon physical exam, she was on a ventilator, resting comfortably. Her vitals were as follows: a blood pressure of 113/60 mmHg, a pulse of 108 beats per minute (bpm), a temperature of 98.0°F, a respiratory rate of 20 breaths per minute (bpm), and a pulse oximeter of 99% on 60% Fi02. The patient had expiratory wheezes heard bilaterally. The patient’s heart had regular rate and rhythm, with no murmurs, gallops, or rubs heard on auscultation. There was no jugular venous distension or peripheral edema noted in all extremities.

After admission, a computed tomography angiography (CTA) was done to rule out a possible pulmonary embolism. Results were negative, along with cardiac workup. The immunoglobulin epsilon (IgE) levels were within reasonable limits. The hematology results showed a significantly high white blood cell (WBC) count with neutrophil predominance, as shown in Table 1.

The patient was sent to the university hospital and seen by an immunologist and allergist. She was given Daliresp (roflumilast) along with azithromycin. Upon medication, symptoms significantly improved with no further acute asthma episodes requiring outpatient and hospitalized visits. 

**Table 1:** Hematology results.

Collected	Result	Units	Reference range
White blood cell	14.1	X 10^3^	4.8-10.8
Hemoglobin	10.3	X10^6^	12.0-16.0
Hematocrit	32.2	%	37.0-47.0
Red blood cell	3.64	X10^6^	4.20-5.40
Platelet	257	X10^3^	130-400
Neutrophils count	10.1	X10^3^	1.5-7.1
Eosinophil count	0.0	X10^3^	0.0-8.0
Basophil count	0.0	X10^3^	0.0-0.1
Lymphocyte count	2.4	X10^3^	0.7-4.3

## Discussion

Once the diagnosis of acute asthma exacerbation is established and the comorbidities and confounders are excluded, the European respiratory society (EUS) and American thoracic society (ATS) define severe asthma as patients treated with high dose inhaled corticosteroids and a bronchodilator or systemic corticosteroid, but their asthma remains uncontrolled despite treatment [5]. Inflammation has a critical role in the pathophysiology of asthma. Immune cells and other immune mediators cause bronchiolar constriction and decrease the diameter of airflow, causing expiratory wheezing, cough, and shortness of breath. Currently, the endotype of asthma is divided into Th2 high and Th2 low inflammation. The standard approach to phenotyping of asthma is based on clinical presentation, associated triggers in a demographic area, and pathological factors. The phenotypical differentiation is not only significant to recognize the disease, but it also helps to follow the response to the medication. The new techniques include biological and specific organ sampling along with the use of inflammatory markers help to differentiate asthma phenotypes. For example, based on the phenotypical presentation, asthma is divided into eosinophilic and noneosinophilic asthma. Eosinophilic asthma has a slow, gradual onset with a slow but effective response to treatment. Neutrophilic asthma occurs rapidly with a quick response to treatment, but patients tend to relapse quickly [4-9].

Th2-high endotype upregulates the expression of T helper type 2 cluster designation (CD) 4+ lymphocytes, leading to the stimulation of cytokines like interleukin (IL) 4, IL5 and IL-13, causing an increase in eosinophil counts in the sputum and airway. The Th2-high endotype eosinophilic asthma is often responsive to steroid therapy. The majority of treatment for asthma is aimed at targeting type 2 inflammation and is directed toward the Th2-High endotype. Anti-IgE therapy was the first effective monoclonal antibody against allergic asthma and given to a patient whose serum IgE level ≥ 30 international unit/milliliters (IU/ml). Presently, two anti-IL5 agents such as mepolizumab and reslizumab have been approved for use in severe eosinophilic asthma. IL5 receptor inhibitors, reslizumab have shown to decrease exacerbation in eosinophilic phenotype asthma. Also, anti-IL-4 receptor alpha therapy like dupiumab has shown to reduce severe asthma exacerbations and has a more significant benefit in patients with higher eosinophil levels [10]. Other medicines that are currently under experiments include anti-thymic stromal lymphopoietin such as tezeplumab, which has shown improvements of severe asthma symptoms in phase IIb clinical trial. The anti-IL-13 antibody tralokinumab has shown inconsistent results in phase III trials, indicating no benefit in treating severe asthma symptoms [11-12].

The pathogenesis of severe acute asthma has not been well established, but bronchial biopsies showed the presence of neutrophilia. The prominent neutrophil counts were also seen in patients with fatal sudden-onset asthma, and they might play an important role in exacerbating asthma. Corticosteroid-resistant asthma mainly is present in adults and is associated with comorbid conditions such as obesity, smoking, low vitamin D levels, and are noneosinophilic (low-Th2 inflammation). The Th2-low endotype does not have any specific biomarker available and is often diagnosed by the exclusion of Th2-high biomarkers. The Th2-low endotype manifests with increased neutrophils and pauci-granulocytic profile. Sur et al. concluded that fast-onset asthma has excess neutrophils relative to eosinophils in the airway submucosa [6] The recruitment of neutrophils involves multiple pathways such as tumor necrosis factor, IL-1, IL-6, IL-8, IL-23, and IL-17 [13].

Currently, there is no specific treatment established for Th2-low asthma. Inhaled steroids can block epithelial IL-8 secretion. However, in a patient with severe asthma, IL-8 concentrations remain significantly high despite a higher dosage. The long-term use of steroids inhibits neutrophil apoptosis which may lead to increasing the neutrophilic count. This may be the reason behind why neutrophil-predominant asthma is resistant to corticosteroids and conventional therapy. The release of IL-8 from neutrophils in the airway leads to an increase in recruitment and activation of neutrophils in the submucosa via C-X-C chemokine receptor 2 (CXCR2). A conclusion of a small double-blinded study done by Todd et al. stated that dual C-X-C chemokines receptors 1 (CXCR1) and CXCR2, antagonists showed a decrease in the amount of circulatory and airway neutrophils via inhibiting its migration [13]. A phase 2 study showed that CXCR2 antagonist SCH527123 is safe and decreases the sputum neutrophils and improves severe asthma symptoms. Macrolides, such as clarithromycin and azithromycin also modulate IL-8 levels along with neutrophil accumulation and activation. It can be a long-term add-on with conservative therapy in a patient with noneosinophilic asthma. However, clinicians should be aware of antibiotic-resistance and its side effects [5, 14-16]. A selective blocker of phosphodiesterase-4 such as roflumilast has shown an improvement in ovalumin-asthmatic mice when combined with fluticasone by decreasing the eosinophils, neutrophils, and macrophage counts in bronchoalveolar lavage fluid [17].

Meanwhile, another clinical trial using CXCR2 antagonist AZD5069 as add-on therapy has shown no significant improvement in patients with severe asthma. The studies using anti-tumor necrosis factor-alpha antibodies for asthma was inconclusive regarding efficacy and had substantial side effects such as infection and malignancy [13, 16]. A randomized control trial of patients with severe asthma treated with the human monoclonal antibody that binds to IL-17 receptor alpha (IL-17Ra) such as brodalumab did not show significant improvement in asthma control. However, the trial was stopped due to side effect such as mental health problem such as suicide. The other IL-17Ra such as secukinumab also did not influence sputum neutrophil counts from baseline in healthy individuals [15, 18]. There are limited treatment options available for Th2-low endotype asthma, and further studies are needed to understand the underlying mechanism and find proper treatment.

## Conclusions

Inhaled steroid and bronchodilator or systemic steroid therapy is inadequate for the Th2-low, neutrophil-predominant endotype of asthma. There are currently no specific treatment guidelines for Th2-low endotype asthma with resistance to available treatment. Recent new medications are explicitly targeting the Th2-high endotype or eosinophilic asthma. The role of specific inflammatory mediators that present in asthma requires an accurate disease sub-phenotyping to optimize the management and to develop an appropriate therapy. For severe and acute-onset asthma resistant to available treatment, understanding that they may have the Th2 low-inflammation endotype and knowing some treatment alternatives for this type of asthma might help prevent recurrent ICU admissions and possibly death. 
